# Survey for positively selected coding regions in the genome of the hematophagous tsetse fly *Glossina morsitans* identifies candidate genes associated with feeding habits and embryonic development

**DOI:** 10.1590/1678-4685-GMB-2018-0311

**Published:** 2020-06-10

**Authors:** Lucas Freitas, Rafael D. Mesquita, Carlos G. Schrago

**Affiliations:** 1 Universidade Federal do Rio de Janeiro, Departamento de Genética, Rio de Janeiro, RJ, Brazil; 2 Universidade Federal do Rio de Janeiro, Instituto de Química, Departamento de Bioquímica, Laboratório de Bioinformática, Rio de Janeiro, RJ, Brazil; 3 Instituto Nacional de Ciência e Tecnologia em Entomologia Molecular, Rio de Janeiro, RJ, Brazil

**Keywords:** Tsetse fly, comparative genomics, Diptera, adaptive molecular evolution, neglected tropical disease

## Abstract

Tsetse flies are responsible for the transmission of *Trypanossoma* sp. to vertebrate animals in Africa causing huge health issues and economic loss. The availability of the genome sequence of *Glossina morsitans* enabled the discovery of several genes related to medically important phenotypes and novel physiological features. However, a genome-wide scan for coding regions that underwent positive selection is still missing, which is surprising given the evolution of traits associated with the hematophagy in this lineage. In this study, we employed an experimental design that controlled for the rate of false positives and we performed a scan of 3,318 *G. morsitans* genes. We found 145 genes with significant historical signal of positive selection. These genes were categorized into 18 functional classes after careful manual annotation. Based on their attributed functions, we identified candidate genes related with feeding habits and embryonic development. When our results were contrasted with gene expression data, we confirmed that most genes that underwent adaptive molecular evolution were frequently expressed in organs associated with key physiological evolutionary innovations in the *G. morsitans* lineage, namely, the salivary gland, the midgut, fat body tissue, and in the spermatophore.

## Introduction

The Glossinidae consists of an African family of flies known as tsetse, which are the vectors responsible for the transmission of *Trypanossoma* sp. to humans and other vertebrates. In humans, trypanosomiasis is known as sleeping sickness, whereas a pathologic condition dubbed ‘nagana’ is reported in other vertebrates (Büscher *et al.*, 2017). These flies are naturally distributed all over the rural areas of sub-Saharan Africa, being endemic to 36 territories, where approximately 70 million people are at risk (Simarro *et al.*, 2012).

In addition to its medical importance, this neglected tropical disease is also responsible for an annual loss of billions of dollars for the livestock industry, as a consequence of diseased farm animals, making animal husbandry extremely hard in infected areas (Kristjanson *et al.*, 1999). Many efforts are being undertaken to overcome trypanosomiasis. In 2009, for the first time in 50 years, the number of new reports in humans dropped to < 10,000 cases, and this trend has been kept steady due to WHO efforts in committed areas (WHO, 2017a). The genome of the tsetse fly *Glossina morsitans* was finished in 2014 (International Glossina Genome Initiative, 2014), and subsequent works identified the function of a number of genes in this species (Benoit *et al.*, 2014a,b, 2015, 2017; Christoffels *et al.*, 2014).

However, although genome sequencing was completed, no genome-wide analysis of positive selection was ever carried out on *G. morsitans*, in order to gain insights on historical signatures of adaptive molecular evolution. These scans are an essential step in comparative genomics analytical pipelines, and they are possible by the increase in the availability of genomes of closely related species (Ellegren, 2014). These genome-wide searches are also relevant to understand the origin of evolutionary innovations, as positively selected genes (PSG) are likely associated with the emergence of novel phenotypes (Nielsen, 2005), which are frequently associated with adaptive response to new environmental conditions, enabling changes of habit/lifestyle. For instance, recent studies have reported positive selection on genes associated with the emergence of eusociality in Hymenoptera (Zhou *et al.*, 2015), the evolution of hypoxia in marine mammals (Foote *et al.*, 2015) and adaptation to carbohydrate diet in humans (Pontremoli *et al.*, 2015).

Two key physiological traits of *G. morsitans* require major evolutionary transitions, namely, viviparity and the blood-feeding (hematophagous) habit. The former is an unusual pattern of reproduction in Diptera, while the latter arose independently several times in different animals, e.g., insects, annelids, and vertebrates (Azar and Nel, 2012). There are two major compatible hypotheses to explain the evolution of blood-feeding habit in insects: the result of a prolonged association with vertebrates, or a morphological pre-adaptation for piercing (Lehane, 2005). Understanding the evolutionary mechanisms that allowed the evolution of hematophagy in insects is important because most hematophagous species are vectors of several diseases (WHO, 2017b).

It has been proposed that hematophagy evolved independently more than ten times in Insecta (Azar and Nel, 2012), which is indicative of the adaptive value of this feeding habit. Despite its importance, few studies have addressed the evolution of this trait using genomic data (Neafsey *et al.*, 2015; Papa *et al.*, 2017). Furthermore, statistical tests implemented were not able to identify lineage-specific positively selected amino acid sites in hematophagous insects yet, which can be later used in advancing new strategies to control these insect vectors (Anisimova, 2015).

In this sense, *G. morsitans* represents an excellent case-study, as it exhibits exclusive characteristics, like hematophagy and viviparity, which are not shared with *Drosophila melanogaster*, its closest sister-lineage with available genome sequence. Therefore, in this study, by performing a comparative evolutionary analysis using an experimental design to control for the rate of false positives, we disclosed genes that evolved under positive selection in the *G. morsitans* lineage alone.

## Material and Methods

### Data preparation

**Figure 1:**
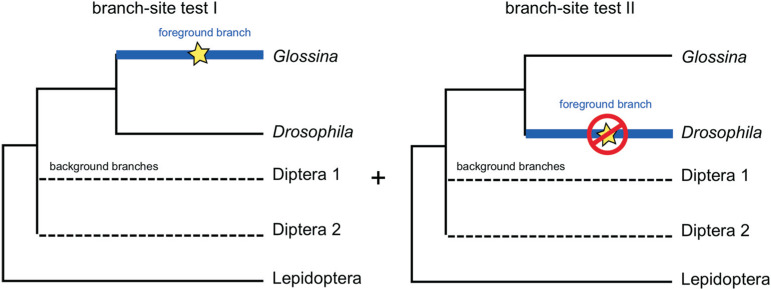
Two-step test which was used to cross-validate positive selection analysis. In Test I, the branch-site test 2 implemented in PAML was used to identify genes (and their respective codon sites) that underwent positive selection in the branch leading to *Glossina* (foreground branch). In Test II, the same procedure was implemented in the non-hematophagous *Drosophila* lineage. We eliminated all genes that were inferred as positively selected in both lineages to gather the set exclusive to *Glossina*.

We used publicly available protein coding sequences of seven Diptera and five Lepidoptera species from different databases (Table S1). We focused on 1:1 orthologous coding regions annotated in OrthoDB v.8 (Kriventseva *et al.*, 2014). Each orthologous group was aligned using PAGAN v. 0.56 (Löytynoja *et al.*, 2012) with default parameters. We applied a custom sequence quality filter to remove sequences shorter than half the average sequence length in each orthologous group. Subsequently, we excluded orthologous groups containing fewer than five species. We established that the minimum species sampling consisted of four Diptera species, which were used to increase the power and specificity of our analysis. Tree rooting was accomplished by employing a Lepidoptera species as outgroup (Figure 1). Because we aimed to identify genes related with hematophagy and embryogenesis evolving under positive selection exclusively in *G. morsitans*, our experimental design included *D. melanogaster*, the sister-lineage in which such traits are absent, in all orthologous groups analyzed. Our final dataset consisted of 3,318 alignments of orthologous coding regions.

### Evolutionary analysis and experimental design

We inferred the gene tree of each orthologous group using RAxML 8.1.17 (Stamatakis, 2014) under the GTR+Γ model (Yang, 1996). Standardization of the substitution model was implemented because GTR is the model that best fits most genes (Ranwez *et al.*, 2007), the same rationale was previously employed by Douzery *et al.* (2014). We used a likelihood ratio test, the Branch-site test 2 (Zhang *et al.*, 2005), as implemented in the codeML program of the PAML 4.8 package (Yang, 2007), to identify amino acid (codon) sites evolving under positive selection in the *G. morsitans* lineage alone. Positive selection indicates that natural selection favored nucleotide changes leading to new amino acids (and possibly new functions) in protein sequences; it is inferred when the rate of non-synonymous substitution (*dN*) surpasses the synonymous substitution rate (*dS*), i.e., *dN*/dS > 1 (Yang and Bielawski, 2000).

While conducting the branch-site tests, branches in the phylogeny were divided into foreground and background lineages, where the foreground lineage was allowed to have sites evolving under positive selection. To control for the rate of false positives, we applied a false discovery rate (FDR) approach to multiple testing (Benjamini and Hochberg, 1995). For each of the 3,318 alignments investigated, we performed two independent tests: one assigning the branch leading to *G. morsitans* as the foreground lineage, and another assigning the branch leading to *D. melanogaster* as the foreground. Therefore, we conducted a total of 6,636 tests in which the FDR correction was applied. A positively selected gene was only deemed as exclusive to the *G. morsitans* lineage if no positive selected codon site was inferred in that same alignment when *D. melanogaster* was used as a foreground lineage (Figure 1). Therefore, if a given coding region was inferred to have undergone positive selection in Tests I and II on different codon sites, it was also discarded. This two-step experimental design was adopted as a methodological cross-validation approach to avoid false positives.

Positively selected codon sites were inferred according to the Bayes empirical Bayes (BEB) approach developed by Yang (2005). If the BEB approach inferred a gene with at least one site with posterior probability > 95% of belonging to the *dN*/*dS* > 1 class, we considered that this gene evolved under positive selection. Because of the large extent of the divergence times between species studied (Misof *et al.*, 2014), we expect the presence of a high degree of saturation of synonymous sites that may lead to errors in the alignments. This issue will impact the quality of the alignment, but not the inference of positive selection (Fletcher and Yang, 2009). Hence, we included another precautionary step, by manually excluding genes in which the positively selected site was located in low-quality alignment regions. We also excluded positively selected codon sites that coded for serine, because such substitutions may incorrectly be identified to be under positive selection, increasing the rate of false-positives (Yang and dos Reis, 2011). Due to the several filters applied, we expect our analytical procedure to be conservative, reducing the type I error rate. Thus, we preferred a statistical framework in which the type II error rate might have increased, instead of providing over credible evidence of molecular adaptation.

### Manual functional annotation and comparison with expression data

While surveying gene functions related with hematophagy and embryonic development, we performed extra checks of the functions of annotated coding regions relying on multiple databases: FlyBase (Gramates *et al.*, 2017), PantherDB (Mi *et al.*, 2017), PFAM (Finn *et al.*, 2016), InterPro (Finn *et al.*, 2017), UniProt (The UniProt Consortium, 2016) and NCBI databases (NCBI Resource Coordinators, 2017). Additionally, we used gene expression data from several works, performed on different tissues (Lehane *et al.*, 2003; Attardo *et al.*, 2006; Alves-Silva *et al.*, 2010; Telleria *et al.*, 2014; Scolari *et al.*, 2016; Bing *et al.*, 2017), to verify the presence of the estimated PSGs. In some works, the list of expressed genes was not available with the proper VectorBase gene ID (Lehane *et al.*, 2003; Attardo *et al.*, 2006; Alves-Silva *et al.*, 2010). We therefore used the IDs available in the GM-s1-Web.xls table of (Alves-Silva *et al.*, 2010) to download all these sequences and then, performed a BLAST v. 2.2.31 (Altschul, 1990) search against the new annotation. The correspondence between both IDs was established and the information available on gene expression was used to detect the presence of PSGs in each tissue-specific expression data.

## Results

**Figure 2:**
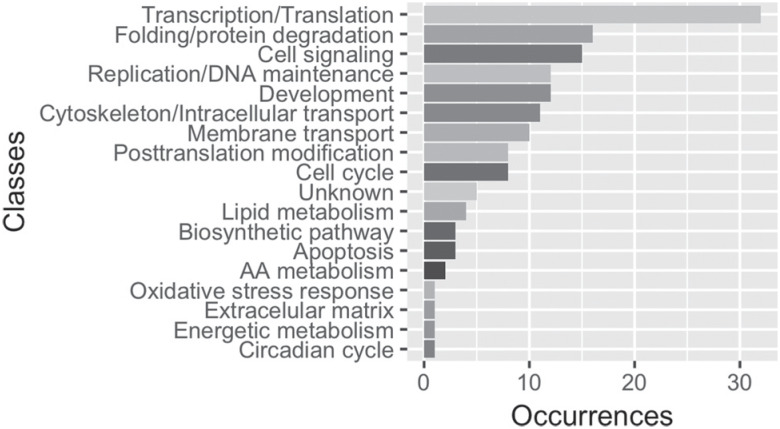
Distribution of positively selected genes exclusive to *Glossina morsitans* according to their functional class.

Using the branch-site test, we were able to identify 145 genes with at least one codon site evolving under positive selection exclusively in *G. morsitans* (Table S2). The manual functional annotation of these PSGs categorized 140 genes into 18 categories (Figure 2, Table S2). The largely recovered categories were transcription/translation (32), folding/protein degradation (16), cell signaling (15), development (12) and replication/DNA maintenance (12).

After annotation, 12 genes were identified as related with embryonic development (Table S2), as they were attributed to the class *development*. Regarding coding regions putatively associated with hematophagy, we identified six genes as potential candidates related with this complex trait due to their role on amino acid metabolism and stress response. Genes GMOY005584 and GMOY010949 participate in alanine-glycine transamination and tryptophan degradation respectively; whereas GMOY004385 is an amino acid transmembrane transport protein. GMOY002159 and GMOY004064 are involved in DNA repair, and GMOY009602 acts on the oxidative stress response. One gene, GMOY005584, is worthy of attention due to its dependence of vitamin B6 produced by the endosymbiont *Wigglesworthia glossinidia* (Michalkova *et al.*, 2014).

Furthermore, two major cellular processes stood out among PSGs, cell maintenance and DNA-mRNA-protein-function balance. Annotation classes (Table S2) included in the cellular processes were: replication/DNA maintenance (12 genes), apoptosis (3), and cell cycle (8); while those associated with protein function were: transcription/translation (32, of which 7 are transcription factors), post-translational modification (8), and protein folding degradation (16).

After comparison with tissue-specific gene expression data, most coding regions were inferred to have undergone adaptive evolution: 109 out of 145 were expressed in organs associated with key evolutionary innovations (Table S3), from which 18 were exclusively found in the salivary glands, 16 in the midgut, 12 in the fat body, and two genes (GMOY011979 and GMOY005584) in the spermatophore.

## Discussion

We were able to identify a total of 145 genes evolving under positive selection in the *G. morsitans* lineage, and our manual annotation identified several candidate genes related to embryonic development and feeding habit. Furthermore, functional classes that were associated with these phenotypes were well represented among PSGs. For instance, genes assigned to protein folding/degradation (16 genes), replication/DNA maintenance (12), cell cycle (8), and apoptosis (2) categories, presumptively deal with the oxidative stress caused by the free heme group during blood-digestion (Sterkel *et al.*, 2017).

Our experimental design was structured in order to control for false positives that may arise when comparing sequences separated by several million years. Adaptive molecular evolution is ultimately inferred by comparing non-synonymous and synonymous distances. Because synonymous sites evolve faster than non-synonymous sites, saturation of evolutionary distance is more likely to impact synonymous changes, decreasing the accuracy of the branch-site test (Gharib and Robinson-Rechavi, 2013). To reduce this effect, we conducted a cross-validation hypothesis testing, applying false discovery rate and carefully filtered gap-rich regions in the alignment. As a result, we expect that the type I error was undermined, despite the evolutionary distances between the species used in our dataset.

The adaptive mutations that gave rise to hematophagy in the *G. morsitans* lineage were fixed in the ancestor of the lineage. Therefore, although a single Calyptratae genome (*G. morsitans*) was investigated, we expect that, when compared to *Drosophila*, substitutions associated with the evolution of these complex traits were correctly inferred along the branch leading to *G. morsitans*. Evidently, in order to single out those substitutions exclusively associated with the emergence of hematophagy, further experimental analyses are required. Especially, the analysis of non-hematophagous species that are evolutionarily closer to *Glossina* should bring further statistical sensitivity to the inference of PSGs associated with this complex trait. We argue, however, that contrasting our results against gene expression data from key tissues is a promising first step in order to gather a set of candidate genes by bioinformatics.

The expression of PSGs in the four tissues where high-throughput data was available (Lehane *et al.*, 2003; Attardo *et al.*, 2006; Alves-Silva *et al.*, 2010; Telleria *et al.*, 2014; Scolari *et al.*, 2016) indicated that 66% (96 out of 145 PSGs) were expressed in the salivary glands and/or midgut tissues. We argue that this provides further corroboration and insights on the putative association of our set of PSGs with hematophagy, as these tissues are clearly involved in the feeding success by their hemostasis counteraction (Ribeiro *et al.*, 2010) and blood digestion.

The salivary gland (Alves-Silva *et al.*, 2010; Telleria *et al.*, 2014) was the organ with the largest number of PSGs (82 out of 145). Previous studies have reported that, in insects, salivary gland genes are under strong selective pressure (Arcà *et al.*, 2014; Neafsey *et al.*, 2015). Notwithstanding, most of those salivary gland PSGs are species-specific, and originated by gene duplications (Ribeiro and Francischetti, 2003; Ribeiro *et al.*, 2010). The amino acid replacements in the 82 PSGs estimated here likely consist of evolutionary innovations against hemostasis, and reinforce that the selective pressure for the effectiveness of the saliva proteins is indeed high in blood-feeding insects.

Recently, Bing *et al.* (2017) analyzed the *G. morsitans* midgut transcriptome. This organ harbors the obligate endosymbiont *W. glossinidia*, which is required for *G. morsitans* reproduction. Midgut transcriptomes were sequenced under three different conditions: the control (with *W. glossinidia*), aposymbiotic,, and infected (with *Trypanosoma brucei rhodesiense*). Compared with the control, five PSGs found here were up-regulated (GMOY000234, GMOY003765, GMOY007531, GMOY008484 and GMOY011346), while one gene was down-regulated (GMOY004385) in both aposymbiotic and infected flies (Table S4). The PSG GMOY009189 was down-regulated only in aposymbiotic flies (Table S4). When control and aposymbiotic flies were compared, 18 PSGs were not differentially expressed, while nine were underexpressed and seven were overexpressed in aposymbiotic flies (Table S4). Among these nine underexpressed genes, GMOY005584 may be a good candidate for understanding the evolution of host-microbiome interaction, as it uses the pyridoxal 5’-phosphate (vitamin B6) produced by its endosymbiont as a cofactor (Michalkova *et al.*, 2014).

It is worth mentioning that the positive selection analysis carried out here was restricted to single copy orthologs. The evolution of a complex trait, such as hematophagy, may be related with evolutionary novelties not directly linked to substitutions in 1:1 orthologs. For instance, the expansion/contraction of gene families, as well as the neofunctionalization of genes, have also been linked to the emergence of adaptive traits (Assis and Bachtrog, 2013; Seppey *et al.*, 2019). Further analyses are required to assess the role of those alternative modes of genome evolution played in the evolution of hematophagy. Such investigation can be implemented using the same experimental design proposed here to control for false positives.

For drawing a comprehensive scenario of the molecular changes that allowed *Glossina* flies to feed on blood, additional comparative genomic data is required. Genome sequencing of non-hematophagous lineages closely related to *Glossina* will increase the accuracy of evolutionary analyses. Moreover, transcriptomes contrasting gene expression under different feeding regimes (blood *vs*. unfed/sugar) in the salivary gland and/or midgut are needed to further investigate the functional role of PSGs in the blood-feeding habit. Also, gene expression profiles from different embryonic stages of development are desirable. The availability of those comparative data will clarify which genes were associated with the rise of physiological innovations in the *G. morsitans* lineage, potentially helping with the development new vector control strategies (Chen *et al.*, 2004; Zhang *et al.*, 2013; Anisimova, 2015).

## Supplementary material

The following online material is available for this article:

Table S1 Species used in this study and the source of the genomes.

Table S2 Manual annotation of the 145 positively selected genes.

Table S3 Distribution of positively selected genes in tissues.

Table S4 Positively selected genes differentially expressed between control and aposymbiotic/Trypanosome infected fly midguts.
