# Supplementary material for: Survey for positively selected coding regions in the genome of the hematophagous tsetse fly Glossina morsitans identifies candidate genes associated with feeding habits and embryonic development
Source: Genet Mol Biol. 2020 Jun 10;43(2):e20180311. doi: 10.1590/1678-4685-GMB-2018-0311 (PMC7288665; doi:10.1590/1678-4685-GMB-2018-0311)
Supplement: Supplementary file 1 [file 1415-4757-GMB-43-2-e20180311-suppl1.pdf]

Supplementary Material to "Survey for positively selected coding regions in the genome of the hematophagous tsetse fly *Glossina morsitans* identifies candidate genes associated with feeding habits and embryonic development"

Table S1 - Species used in this study and the source of the genomes.

Species	Database	Reference
Anopheles gambiae	VectorBase	(Giraldo-Calderon *et al.*, 2014)
Aedes aegypti	VectorBase	(Giraldo-Calderon *et al.*, 2014)
Culex quinquefasciatus	VectorBase	(Giraldo-Calderon *et al.*, 2014)
Lutzomyia longipalpis	VectorBase	(Giraldo-Calderon *et al.*, 2014)
Glossina morsitans	VectorBase	(Giraldo-Calderon *et al.*, 2014)
Drosophila melanogaster	FlyBase	(Gramates *et al.*, 2016)
Ceratitis capitata	i5K	(i5K Consortium, 2013)
Bombyx mori	SilkDB	(Wang *et al.*, 2005)
Manduca sexta	Agripest Base	(Brown and Caragea, 2017)
Plutella xylostella	DBM-DB	(Tang *et al.*, 2014)
Danaus plexippus	MonarchBase	(Zhan and Reppert, 2013)
Heliconius melpomene	Butterfly Genome Database	(Reed and Pillardy, 2017)

References

Brown SJ and Caragea D (2017) Agricultural Pest Genomics Resources [WWW Document]. URL http://www.agripestbase.org (accessed 23 November 2017).

Giraldo-Calderon GI, Emrich SJ, MacCallum RM, Maslen G, Dialynas E, Topalis P, Ho N, Gesing S, Madey G, Collins FH *et al.* (2014) VectorBase: an updated bioinformatics resource for invertebrate vectors and other organisms related with human diseases. Nucleic Acids Res 43:D707–D713.

Gramates LS, Marygold SJ, dos Santos G, Urbano JM, Antonazzo G, Matthews BB, Rey AJ, Tabone CJ, Crosby MA, Emmert DB *et al.* (2016) FlyBase at 25: looking to the future. Nucleic Acids Res 45:D663–D671.

i5K Consortium (2013) The i5K Initiative: advancing arthropod genomics for knowledge, human health, agriculture, and the environment. J Hered 104:595–600.

Reed B and Pillardy J (2017) Butterfly Genome Database [WWW Document]. URL http://www.butterflygenome.org/ (accessed 23 November 2017).

Tang W, Yu L, He W, Yang G, Ke F, Baxter SW, You S, Douglas CJ and You M (2014) DBM-DB: the diamondback moth genome database. Database 2014:bat087.

Wang J, Xia Q, He X, Dai M, Ruan J, Chen J, Yu G, Yuan H, Hu Y, Li R *et al.* (2005) SilkDB: a knowledgebase for silkworm biology and genomics. Nucleic Acids Res 33:D399–402.

Zhan S and Reppert SM (2013) MonarchBase: the monarch butterfly genome database. Nucleic Acids Res 41:D758–63.
