# Supplementary material for: Survey for positively selected coding regions in the genome of the hematophagous tsetse fly Glossina morsitans identifies candidate genes associated with feeding habits and embryonic development
Source: Genet Mol Biol. 2020 Jun 10;43(2):e20180311. doi: 10.1590/1678-4685-GMB-2018-0311 (PMC7288665; doi:10.1590/1678-4685-GMB-2018-0311)
Supplement: Supplementary file 2 [file 1415-4757-GMB-43-2-e20180311-suppl2.pdf]

Supplementary Material to "Survey for positively selected coding regions in the genome of the hematophagous tsetse fly *Glossina morsitans* identifies candidate genes associated with feeding habits and embryonic development"

Table S2 - Manual annotation of the 145 positively selected genes.

G. morsitans ID	class	gene	Cofactor
GMOY005584	AA metabolism	Alanine--glyoxylate aminotransferase	pyridoxal 5'-phosphate
GMOY010949	AA metabolism	cinnabar (Kynurenine 3-monooxygenase)	FAD
GMOY005494	Apoptosis	phospholipid-translocating P-type ATPase, flippase	-
GMOY005973	Apoptosis	Death regulator Nedd2-like caspase	-
GMOY006388	Apoptosis	Kibra	-
GMOY011622	Biosynthetic pathway - glicolipids	beta4GalNAcTB (Golgi glycosyltransferase that transfers N-acetylgalactosamine to N-acetylglucosamine residues of glycosphingolipids)	-
GMOY007069	Biosynthetic pathway - heme	Protoporphyrinogen oxidase	FAD
GMOY008796	Biosynthetic pathway - isoprenoid	isopentenyl-diphosphate delta-isomerase	-
GMOY000573	Cell cycle	Anaphase Promoting Complex subunit 4 [Source:FlyBase;Acc:FBgn0052707]	-
GMOY002211	Cell cycle	Cyclin	-
GMOY003790	Cell cycle	misato (mitotic spindle assembly)	-
GMOY006171	Cell cycle	CWF19, CELL CYCLE CONTROL PROTEIN	-
GMOY006906	Cell cycle	Centrosomal protein 97kDa	-
GMOY009036	Cell cycle	Asterless	-
GMOY010307	Cell cycle	Cyclin Y [Source:Projected from Drosophila melanogaster (FBgn0032378) FlyBase gene name;Acc:FBgn0032378]	-
GMOY011635	Cell cycle / protein secretion	MLF1-adaptor molecule	-
GMOY001576	Cell signaling	phosphatidylinositol-4-phosphate 3-kinase [Source:VB Community Annotation]	-
GMOY002379	Cell signaling	Phosphatidylinositol 4,5-bisphosphate 4-phosphatase	-
GMOY002994	Cell signaling	Snmp1 - Sensory neuron membrane protein 1	-

***G. morsitans* ID**	**class**	**gene**	**Cofactor**
GMOY004274	Cell signaling	Adenylyl cyclase 35C	-
GMOY004900	Cell signaling	Secretion-associated Ras-related 1	-
GMOY006177	Cell signaling	Octopamine receptor in mushroom bodies	-
GMOY006886	Cell signaling	Ras GTPase activating protein 1	-
GMOY008361	Cell signaling	Ac3 (adenylate cyclase)	Mg2+
GMOY008484	Cell signaling	Protein kinase, cAMP-dependent, regulatory subunit type 2	-
GMOY010429	Cell signaling	Protein phosphatase 2C	-
GMOY010513	Cell signaling	Rho GTPase activating protein at 1A	-
GMOY010713	Cell signaling	Plexin A	-
GMOY011192	Cell signaling	Myopic (PROTEIN TYROSINE PHOSPHATASE)	-
GMOY009428	Cell signaling / phospholipid transport	Vibrator	-
GMOY005612	Cell signaling / protein degradation	Phospholipase A2 activator protein	-
GMOY002026	Circadian cycle	SRR1	-
GMOY003765	Cytoskeleton/Intracellular transport	Moesin	-
GMOY005981	Cytoskeleton/Intracellular transport	spindle pole body protein	-
GMOY005983	Cytoskeleton/Intracellular transport	Garnet	-
GMOY006635	Cytoskeleton/Intracellular transport	sorting nexin-29	-
GMOY007246	Cytoskeleton/Intracellular transport	Sac1 phosphatase	-
GMOY008208	Cytoskeleton/Intracellular transport	Sec7-protein (golgi biogenesis)	-
GMOY008690	Cytoskeleton/Intracellular transport	didum (dilute class unconventional myosin)	-
GMOY010091	Cytoskeleton/Intracellular transport	Yip1 interacting factor (integral membrane protein required for membrane fusion of ER derived vesicles)	-
GMOY010215	Cytoskeleton/Intracellular transport	Px protein	-
GMOY010861	Cytoskeleton/Intracellular transport	asrij (Ovarian carcinoma immunoreactive antigen)	-
GMOY005058	Cytoskeleton/Intracellular transport/Development	Neurexin IV	-
GMOY005210	Development	Nodal modulator 1 (Carbohydrate binding / membrane)	-
GMOY005538	Development	Zinc finger HIT domain-containing protein (neurogenesis)	-
GMOY009946	Development	draper (membrane receptor involved in phagocytosis)	-

***G. morsitans* ID**	**class**	**gene**	**Cofactor**
GMOY008890	Development - cilia	TRAF3-interacting protein 1 (cilia assembly / microtubule binding)	-
GMOY008382	Development - cilia assembly	ARL2_Bind_BART protein	-
GMOY007795	Development - egg and dorsoventral / posttranslation modification	nudel (protease)	-
GMOY007252	Development - muscle	flightless I (actin binding)	-
GMOY007401	Development - ocelli	reduced ocelli	-
GMOY011979	Development - trachea	Vacuolar H+-ATPase 26kD subunit	-
GMOY007531	Development - trachea /Cell signaling	Windpipe	-
GMOY007724	Development / cilia movement	DPCD protein	-
GMOY008836	Development /Cell signaling	Son of sevenless	-
GMOY009188	Energetic metabolism	thymidylate/uridylate kinase (dNTPs synthesis)	-
GMOY007883	extracelular matrix	Papilin	-
GMOY000429	Folding/protein degradation	E3 UBIQUITIN-PROTEIN LIGASE HAKAI	-
GMOY000601	Folding/protein degradation	OTU-like cysteine protease	-
GMOY001329	Folding/protein degradation	Chaperona DnaJ	-
GMOY003507	Folding/protein degradation	F BOX AND LEUCINECH REPEAT PROTEIN	-
GMOY004213	Folding/protein degradation	Ubiquitin carboxyl-terminal hydrolase MINDY-3 homolog	-
GMOY005513	Folding/protein degradation	proteasome regulatory particle	-
GMOY006168	Folding/protein degradation	GDI interacting protein 3 (ubiquitin-proteasome system peptide N-glycanase)	-
GMOY006379	Folding/protein degradation	Ubiquitin system component Cue	-
GMOY007360	Folding/protein degradation	burgundy (deubiquitinase activator)	-
GMOY007820	Folding/protein degradation	Puromycin sensitive aminopeptidase	Zn^{2+}
GMOY009591	Folding/protein degradation	Protein disulfide isomerase	-
GMOY009645	Folding/protein degradation	Diablo	-
GMOY010378	Folding/protein degradation	UBX-TUG protein	-
GMOY010921	Folding/protein degradation	metalloendopeptidase - mitocondrial	Zn^{2+}
GMOY011545	Folding/protein degradation	CHCH protein	-
GMOY001096	Folding/protein degradation / Cell cycle	E3 ligase	-

G. morsitans ID	class	gene	Cofactor
GMOY000234	Lipid metabolism	acyl-CoA synthetase [Source:VB Community Annotation]	-
GMOY010585	Lipid metabolism	Palmitoyl-protein Thioesterase 2	-
GMOY010659	Lipid metabolism	adipose [Source:Projected from Drosophila melanogaster (FBgn0000057) FlyBase gene name;Acc:FBgn0000057]	-
GMOY008064	Lipid metabolism - membrane biosynthesis	choline ethanolamine kinase	-
GMOY002640	M: Cell wall/membrane/envelope biogenesis	ext2	Mn2+
GMOY000690	Membrane transport	TWK-35 POTASSIUM CHANNEL, SUBFAMILY K	-
GMOY002117	Membrane transport	cation-P04 transporter	-
GMOY002539	Membrane transport	Syt14 - Synaptotagmin	-
GMOY003261	Membrane transport	Peroxin5 (peroxisome matrix targeting signal-1 binding)	-
GMOY004069	Membrane transport	LETM1-like protein	-
GMOY004385	Membrane transport	amino acid transmembrane transport	-
GMOY007842	Membrane transport	potassium:chloride symporter	-
GMOY008254	Membrane transport	embargoed (nuclear export signal receptor activity)	-
GMOY010031	Membrane transport	Mitochondrial import inner membrane translocase subunit Tim21	-
GMOY009602	oxidative stress response	Peroxidasin	Ca2+, heme b
GMOY000365	Posttranslation modification	galactosyltransferases [Source:VB Community Annotation]	-
GMOY000778	Posttranslation modification	galactosyltransferases [Source:VB Community Annotation]	-
GMOY006033	Posttranslation modification	aminopeptidase P	Mn2+
GMOY006749	Posttranslation modification	pigeon gamma-secretase	-
GMOY006827	Posttranslation modification	tungus N terminal glutamine amidase	-
GMOY007052	Posttranslation modification	IKB kinase	-
GMOY006174	Posttranslation modification / Cell signaling	rhomboid-7 (intramembrane protease)	-
GMOY009189	Posttranslation modification / Cell signaling	Farnesyl transferase alpha	-
GMOY000976	Replication/DNA maintenance	Helicase	-
GMOY001360	Replication/DNA maintenance	Est1 - telomerase activating	-

G. morsitans ID	class	gene	Cofactor
GMOY002159	Replication/DNA maintenance	DNA repair protein	[4Fe-4S] cluster
GMOY003360	Replication/DNA maintenance	DNA methyltransferase	-
GMOY003381	Replication/DNA maintenance	Transcriptional factor OR histone demethylation OR tRNA synthesis	-
GMOY003455	Replication/DNA maintenance	RNA helicase	-
GMOY003531	Replication/DNA maintenance	DNA helicase	-
GMOY004064	Replication/DNA maintenance	rev1	-
GMOY008987	Replication/DNA maintenance	DNA_pol_delta/II_ssu	-
GMOY009115	Replication/DNA maintenance	SmydA-5	-
GMOY010955	Replication/DNA maintenance	methyltransferase BMT2 homolog [Source:UniProtKB/TrEMBL;Acc:A0A1B0GCC6]	-
GMOY008666	Replication/DNA maintenance or cell signalling	endonucleases or phosphatases or phosphodiesterase	-
GMOY006305	Tanscription/Translation/Apoptosis	mitochondrial ribosomal protein S30 (programmed cell death protein 9 PDCD)	-
GMOY000579	Transcription/Translation	Nop52	-
GMOY001272	Transcription/Translation	Ntrap	-
GMOY001405	Transcription/Translation	Bing4 (Homologo do WD46 em humanos, onde este é relacionado a rRNA processin in the nucleus and cytosol)	-
GMOY001952	Transcription/Translation	RNA binding protein	-
GMOY002401	Transcription/Translation	snRNA U1 and U2 transcription associated protein	-
GMOY002632	Transcription/Translation	tho2	-
GMOY003329	Transcription/Translation	60S ribosomal protein L6	-
GMOY004003	Transcription/Translation	rRNA maturation protein	-
GMOY004559	Transcription/Translation	mRNA 3'-UTR binding/splicing protein	-
GMOY004775	Transcription/Translation	fork head	-
GMOY005390	Transcription/Translation	Arginyl-tRNA synthetase	-
GMOY005640	Transcription/Translation	rRNA PROCESSING PROTEIN EBNA1-BINDING PROTEINLATED	-
GMOY005662	Transcription/Translation	Glorund	-
GMOY005984	Transcription/Translation	enhancer of mRNA-decapping protein 3 (EDC3)	-
GMOY006536	Transcription/Translation	DNA_pol_A egalitarian	-

G. morsitans **ID**	**class**	**gene**	**Cofactor**
GMOY006663	Transcription/Translation	sip1 (spliceosome complex)	-
GMOY006741	Transcription/Translation	ribossomal protein Rsm-22-like	-
GMOY006819	Transcription/Translation	JAZ transcriptional factor	-
GMOY008100	Transcription/Translation	ribosomal large subunit biogenesis protein	-
GMOY008282	Transcription/Translation	mitochondrial large ribosomal subunit	-
GMOY009454	Transcription/Translation	Eukaryotic translation initiation factor 3 subunit F [Source:UniProtKB/TrEMBL;Acc:A0A1B0G815]	-
GMOY009683	Transcription/Translation	DNA POLYMERASE V RELATED	-
GMOY009883	Transcription/Translation	tRNA (uracil-O(2)-)-methyltransferase	-
GMOY010001	Transcription/Translation	mitochondrial ribosomal protein L47	-
GMOY010490	Transcription/Translation	U3 small nucleolar ribonucleoprotein protein MPP10	-
GMOY010662	Transcription/Translation	Heat shock factor (transcriptional factor)	-
GMOY011346	Transcription/Translation	CREB3 REGULATORY FACTOR	-
GMOY011513	Transcription/Translation	Translational activator GCN1	-
GMOY011921	Transcription/Translation	PROTEIN PARTNER OF SNF, ISOFORM B	-
GMOY011952	Transcription/Translation	Transcription factor MBF1 [Source:UniProtKB/TrEMBL;Acc:D3TPV9]	-
GMOY009186	Transcription/Translation / others	N-acetyltransferase	-
GMOY001832	Unknown	Unknown	-
GMOY003797	Unknown	osiris 11 (conserved gene, unknown function, integral component of membrane)	-
GMOY008173	Unknown	Arm-protein	-
GMOY008546	Unknown	Unknown	-
GMOY010885	Unknown	Suppressor APC domain-containing protein 2	-
