# Supplementary material for: Survey for positively selected coding regions in the genome of the hematophagous tsetse fly Glossina morsitans identifies candidate genes associated with feeding habits and embryonic development
Source: Genet Mol Biol. 2020 Jun 10;43(2):e20180311. doi: 10.1590/1678-4685-GMB-2018-0311 (PMC7288665; doi:10.1590/1678-4685-GMB-2018-0311)
Supplement: Supplementary file 3 [file 1415-4757-GMB-43-2-e20180311-suppl3.pdf]

Supplementary Material to "Survey for positively selected coding regions in the genome of the hematophagous tsetse fly *Glossina morsitans* identifies candidate genes associated with feeding habits and embryonic development"

Table S3 - Distribution of positively selected genes in tissues.

G. morsitans ID	BLAST score	Alves-Silva *et al.* 2010 ID	Fatbody	Salivary gland	Midgut
GMOY000579	1506	sgmgfb-contig_16503	0	1	0
GMOY004559	115	sgmgfb-contig_16515	0	1	0
GMOY005981	1557	sgmgfb-contig_13589	0	1	0
GMOY006177	139	sgmgfb-contig_16539	0	1	0
GMOY006819	1000	sgmgfb-contig_15764	0	1	0
GMOY009602	556	sgmgfb-contig_14218	0	1	0
GMOY011513	1219	sgmgfb-contig_2684	0	1	0
GMOY001405	1304	sgmgfb-contig_8389	0	2	0
GMOY001832	1639	sgmgfb-contig_8485	0	2	0
GMOY005983	1238	sgmgfb-contig_13534	0	2	0
GMOY009188	483	sgmgfb-contig_16346	0	2	0
GMOY010662	830	sgmgfb-contig_15723	0	2	0
GMOY011192	1151	sgmgfb-contig_14264	0	2	0
GMOY004274	1245	sgmgfb-contig_8063	0	3	0
GMOY006749	1423	sgmgfb-contig_14629	0	3	0
GMOY008208	1807	sgmgfb-contig_5517	0	4	0
GMOY006388	2141	sgmgfb-contig_5514	0	5	0
GMOY000429	1781	sgmgfb-contig_10153	1	0	0
GMOY000778	1188	sgmgfb-contig_9655	1	0	0
GMOY001360	1628	sgmgfb-contig_9873	1	0	0
GMOY003360	1351	sgmgfb-contig_9971	1	0	0
GMOY006635	1395	sgmgfb-contig_9421	1	0	0
GMOY008836	438	sgmgfb-contig_9353	1	0	0
GMOY009645	1712	sgmgfb-contig_9118	1	0	0
GMOY010513	1559	sgmgfb-contig_10000	1	0	0
GMOY011635	1633	sgmgfb-contig_9453	1	0	0
GMOY006379	1648	sgmgfb-contig_6650	1	1	0
GMOY009683	774	sgmgfb-contig_16574	1	1	0
GMOY001096	1914	sgmgfb-contig_7240	1	2	0
GMOY001576	514	sgmgfb-contig_15336	1	2	0
GMOY001952	863	sgmgfb-contig_7222	1	2	0
GMOY010031	2161	sgmgfb-contig_4633	1	4	0
GMOY002632	1179	sgmgfb-contig_7163	2	0	0

G. morsitans ID	BLAST score	Alves-Silva *et al.* 2010 ID	Fatbody	Salivary gland	Midgut
GMOY007842	1454	sgmgfb-contig_6618	2	1	0
GMOY003790	1740	sgmgfb-contig_4838	2	2	0
GMOY010215	2586	sgmgfb-contig_5234	2	2	0
GMOY002379	1995	sgmgfb-contig_4562	3	2	0
GMOY010429	405	sgmgfb-contig_4855	4	0	0
GMOY003455	2780	sgmgfb-contig_4423	5	0	0
GMOY002026	320	sgmgfb-contig_15205	5	1	0
GMOY003261	3175	sgmgfb-contig_3753	5	2	0
GMOY007360	4252	sgmgfb-contig_2894	8	2	0
GMOY002211	785	sgmgfb-contig_10317	0	0	1
GMOY006741	963	sgmgfb-contig_12313	0	0	1
GMOY007883	592	sgmgfb-contig_10318	0	0	1
GMOY002640	843	sgmgfb-contig_14724	0	2	1
GMOY004385	1291	sgmgfb-contig_8202	0	7	1
GMOY002117	2636	sgmgfb-contig_4872	4	0	1
GMOY005984	931	sgmgfb-contig_4430	4	0	1
GMOY009883	2868	sgmgfb-contig_2307	4	7	1
GMOY000573	1079	sgmgfb-contig_13202	0	0	2
GMOY001272	830	sgmgfb-contig_10644	0	0	2
GMOY006886	793	sgmgfb-contig_11603	0	0	2
GMOY010490	1530	sgmgfb-contig_7745	0	0	2
GMOY010659	603	sgmgfb-contig_11514	0	0	2
GMOY004064	1827	sgmgfb-contig_6278	0	1	2
GMOY005538	1110	sgmgfb-contig_7882	0	1	2
GMOY007252	1447	sgmgfb-contig_7721	0	2	2
GMOY010921	1600	sgmgfb-contig_7934	0	2	2
GMOY005210	1434	sgmgfb-contig_6167	0	3	2
GMOY005058	459	sgmgfb-contig_14449	1	1	2
GMOY011346	1912	sgmgfb-contig_7185	1	1	2
GMOY006536	885	sgmgfb-contig_5199	1	3	2
GMOY008484	2446	sgmgfb-contig_3895	1	8	2
GMOY010378	1834	sgmgfb-contig_4869	2	4	2
GMOY010955	1748	sgmgfb-contig_3770	3	2	2
GMOY002401	1365	sgmgfb-contig_16512	4	4	2
GMOY007820	2597	sgmgfb-contig_2957	4	5	2
GMOY008173	3059	sgmgfb-contig_2569	4	7	2
GMOY011952	1825	sgmgfb-contig_3732	5	0	2
GMOY003381	2894	sgmgfb-contig_1863	13	4	2
GMOY006168	2479	sgmgfb-contig_1944	6	8	3
GMOY003531	2451	sgmgfb-contig_8009	0	4	4
GMOY008064	1958	sgmgfb-contig_1914	0	14	4
GMOY005640	1840	sgmgfb-contig_4659	1	0	4

***G. morsitans* ID**	**BLAST score**	**Alves-Silva *et al.* 2010 ID**	**Fatbody**	**Salivary gland**	**Midgut**
GMOY004003	1729	sgmgfb-contig_5145	1	3	4
GMOY010091	1147	sgmgfb-contig_2364	1	9	4
GMOY005612	4519	sgmgfb-contig_2356	2	8	4
GMOY010307	922	sgmgfb-contig_15846	3	2	4
GMOY009428	3123	sgmgfb-contig_2838	3	6	4
GMOY006033	3426	sgmgfb-contig_2403	7	3	4
GMOY008546	577	sgmgfb-contig_15631	13	2	4
GMOY005513	3166	sgmgfb-contig_3910	1	1	5
GMOY000365	2150	sgmgfb-contig_3053	2	3	5
GMOY003765	628	sgmgfb-contig_16171	3	7	5
GMOY010861	2015	sgmgfb-contig_660	5	5	5
GMOY006174	2390	sgmgfb-contig_3382	0	3	6
GMOY010001	1738	sgmgfb-contig_3243	1	2	6
GMOY010713	2863	sgmgfb-contig_3441	2	2	6
GMOY004069	1424	sgmgfb-contig_1909	5	6	6
GMOY006305	3321	sgmgfb-contig_1690	6	7	6
GMOY008282	1821	sgmgfb-contig_1734	9	3	6
GMOY005584	2942	sgmgfb-contig_506	36	5	7
GMOY000234	3278	sgmgfb-contig_493	44	3	7
GMOY006663	706	sgmgfb-contig_6100	0	0	8
GMOY000601	3009	sgmgfb-contig_2633	4	0	8
GMOY008100	2610	sgmgfb-contig_2429	4	2	8
GMOY009454	1912	sgmgfb-contig_1523	10	2	8
GMOY003329	1703	sgmgfb-contig_1743	12	3	8
GMOY007531	2739	sgmgfb-contig_4249	4	3	9
GMOY007246	4174	sgmgfb-contig_1862	3	4	10
GMOY004900	1930	sgmgfb-contig_1421	9	4	11
GMOY010585	1264	sgmgfb-contig_1484	4	5	13
GMOY011979	4735	sgmgfb-contig_934	6	13	13
GMOY009591	4859	sgmgfb-contig_499	28	19	13
GMOY008666	2867	sgmgfb-contig_2499	3	8	14
GMOY010949	1932	sgmgfb-contig_1430	8	0	16
GMOY009189	2202	sgmgfb-contig_1249	4	1	19
GMOY004775	* Telleria *et al.*, (2014)		-	-	-
GMOY011979	** Scolari *et al.*, (2016)		-	-	-
GMOY005584	** Scolari *et al.*, (2016)		-	-	-

*Gene found on salivary gland.

**Genes found on spermatophore.
