# Supplementary material for: Survey for positively selected coding regions in the genome of the hematophagous tsetse fly Glossina morsitans identifies candidate genes associated with feeding habits and embryonic development
Source: Genet Mol Biol. 2020 Jun 10;43(2):e20180311. doi: 10.1590/1678-4685-GMB-2018-0311 (PMC7288665; doi:10.1590/1678-4685-GMB-2018-0311)
Supplement: Supplementary file 4 [file 1415-4757-GMB-43-2-e20180311-suppl4.pdf]

Supplementary Material to "Survey for positively selected coding regions in the genome of the hematophagous tsetse fly *Glossina morsitans* identifies candidate genes associated with feeding habits and embryonic development"

Table S4 - Positively selected genes differentially expressed between control and aposymbiotic/Trypanosome infected fly midguts. Gene expression from Bing *et al.* (2017), Table S6 and S9.

G. morsitans ID	Expression pattern
GMOY000234	Upregulated in aposymbiotic and infected flies
GMOY003765	Upregulated in aposymbiotic and infected flies
GMOY004385	Downregulated in aposymbiotic and infected flies
GMOY007531	Upregulated in aposymbiotic and infected flies
GMOY008484	Upregulated in aposymbiotic and infected flies
GMOY009189	Downregulated in aposymbiotic flies
GMOY011346	Upregulated in aposymbiotic and infected flies
GMOY000365	No Change
GMOY000429	No Change
GMOY000601	Underexpressed in aposymbiotic
GMOY000976	Underexpressed in aposymbiotic
GMOY001576	No Change
GMOY002379	No Change
GMOY002640	No Change
GMOY003455	Overexpressed in aposymbiotic
GMOY003531	No Change
GMOY004064	No Change
GMOY004274	Overexpressed in aposymbiotic
GMOY004900	No Change
GMOY005390	No Change
GMOY005494	Underexpressed in aposymbiotic
GMOY005584	Underexpressed in aposymbiotic
GMOY005973	Overexpressed in aposymbiotic
GMOY006033	Underexpressed in aposymbiotic
GMOY006174	Underexpressed in aposymbiotic
GMOY007052	No Change
GMOY007360	No Change
GMOY007820	No Change
GMOY008064	Overexpressed in aposymbiotic
GMOY008361	No Change
GMOY008666	Underexpressed in aposymbiotic
GMOY008796	No Change

G. morsitans ID	Expression pattern
GMOY009188	Underexpressed in aposymbiotic
GMOY009591	No Change
GMOY009602	Overexpressed in aposymbiotic
GMOY009683	Overexpressed in aposymbiotic
GMOY010429	No Change
GMOY010585	No Change
GMOY010921	Underexpressed in aposymbiotic
GMOY010949	No Change
GMOY011192	Overexpressed in aposymbiotic
